# Neuroprotective effect of Demethoxycurcumin, a natural derivative of Curcumin on rotenone induced neurotoxicity in SH-SY 5Y Neuroblastoma cells

**DOI:** 10.1186/s12906-017-1720-5

**Published:** 2017-04-18

**Authors:** Muthu Ramkumar, Srinivasagam Rajasankar, Veerappan Venkatesh Gobi, Chinnasamy Dhanalakshmi, Thamilarasan Manivasagam, Arokiasamy Justin Thenmozhi, Musthafa Mohamed Essa, Ameer Kalandar, Ranganathan Chidambaram

**Affiliations:** 1 Research Scholar, Bharath University, Selaiyur, Chennai, Tamil Nadu 600073 India; 2 Department of Anatomy, Velammal Medical College and Hospital, Madurai, Tamil Nadu 625009 India; 3 Department of Biochemistry and Biotechnology, Annamalai University, Annamalainagar, Tamil Nadu 608002 India; 4 Department of Food Science and Nutrition, CAMS, Sultan Qaboos University, Muscat, Oman; 5 Ageing and Dementia Research Group, Sultan Qaboos University, Muscat, Oman; 6 Food and Brain Research Foundation, Chennai, Tamil Nadu 600094 India; 7 School of Medicine, College of Health and Medical Sciences, Haramaya University, Dire Dawa, Ethiopia; 8 Department of Radiology, Sri Lakshminarayana Institute of Medical Sciences, Puducherry, India

**Keywords:** Demethoxycurcumin, Rotenone, Oxidative stress, Apoptosis, Neurodegenerative diseases

## Abstract

**Background:**

Mitochondrial dysfunction and oxidative stress are the main toxic events leading to dopaminergic neuronal death in Parkinson’s disease (PD) and identified as vital objective for therapeutic intercession. This study investigated the neuro-protective effects of the demethoxycurcumin (DMC), a derivative of curcumin against rotenone induced neurotoxicity.

**Methods:**

SH-SY5Y neuroblastoma cells are divided into four experimental groups: untreated cells, cells incubated with rotenone (100 nM), cells treated with DMC (50 nM) + rotenone (100 nM) and DMC alone treated. 24 h after treatment with rotenone and 28 h after treatment with DMC, cell viability was assessed using the MTT assay, and levels of ROS and MMP, plus expression of apoptotic protein were analysed.

**Results:**

Rotenone induced cell death in SH-SY5Y cells was significantly reduced by DMC pretreatment in a dose-dependent manner, indicating the potent neuroprotective effects of DMC. Rotenone treatment significantly increases the levels of ROS, loss of MMP, release of Cyt-c and expression of pro-apoptotic markers and decreases the expression of anti-apoptotic markers.

**Conclusions:**

Even though the results of the present study indicated that the DMC may serve as a potent therapeutic agent particularly for the treatment of neurodegenerative diseases like PD, further pre-clinical and clinical studies are required.

**Electronic supplementary material:**

The online version of this article (doi:10.1186/s12906-017-1720-5) contains supplementary material, which is available to authorized users.

## Background

Parkinson’s disease (PD) is one of the most common and progressive neurodegenerative disease, which affects the movement of aged population and is characterized by the selective loss of dopaminergic neurons in the substantia nigra of pars compacta (SNpc) [1] and depletion of dopamine (DA), a neurotransmitter responsible for movement. Though the cause of PD is unknown, abnormal processes such as Lewy body formation, calcium homeostasis, glutamate toxicity, inflammation, proteasome dysfunction and apoptosis are reported to be involved in induction and PD progression. Enhanced mitochondrial dysfunction and its mediated oxidative stress also play a key role in the pathophysiology of PD [2].

Animal and epidemiological studies have indicated that the exposure of pesticide can also increase the risk of PD [3, 4]. Rotenone, a naturally occurring plant flavonoid and well known neurotoxic pesticide, readily crosses the blood-brain barrier due to its high lipophilic nature, transverse the cellular membrane without the need of dopamine transporter. Further, it accumulates and impairs mitochondrial function, mediates oxidative stress and ultimately leads to neurodegeneration [5–7]. Accordingly, a rotenone induced PD model might have various advantages than several other PD models [8].

Levodopa (L-DOPA) remains the most effective treatment for PD. Prolonged treatment of L-DOPA is associated with various side effects and resistance. Although several strategies are developed to manage the disease, the mechanism still remains enigmatic. In recent years, considerable research has been carried out on identifying naturally occurring neuroprotective substances, aimed to prevent or delay the neurodegenerative processes. Turmeric has long been known as a spice, dye and home medicine for jaundice, menstrual difficulties, bloody urine, hemorrhage, toothache, bruises and chest pain [9, 10]. Epidemiological studies by Ganguli et al., [11] have suggested that the consumption of turmeric by the Indian populations is correlated with the low incidence of Alzheimer’s disease (AD)/PD as compared to the Caucasians. The neuroprotective effect of turmeric can be attributed due to the presence of active polyphenol, curcumin which imparts the characteristic color and properties [12]. Both the in vitro and in vivo experiments indicated that the anti-parkinsonic effect of curcumin attributed due to its anti-oxidant [13], mitochondrial protective [14], signal modulating [15], anti-inflammatory [16, 17] and anti-apoptotic properties [18].

**Fig. 1:**
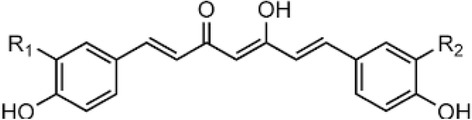
Structure of curcumin, demethoxycurcumin, and bisdemethoxycurcumin. Curcumin: R_1_ = R_2_ = OCH_3_. Demethoxycurcumin: R_1_ = H; R_2_ = OCH_3_. Bisdemethoxycurcumin: R_1_ = R_2_ = H

Other than curcumin, demethoxycurcumin (DMC) (4-hydroxycinnamoyl-(feruloyl) methane) (Fig. 1) and bisdemethoxycurcumin (BMC) (bis(4-hydroxycinnamoyl)methane), are the other curcuminoids mainly present in turmeric. 95% of curcumin as a mixture of three curcuminoids, typically comprising around 77% curcumin, 17% DMC, and 6% BMC [19, 20] were available commercially. Curcumin analogs such as DMC and BMC, have also been reported to possess considerable antioxidant, anti-inflammatory and anti-proliferative activities [21, 22]. Predominantly DMC is reported to have better anticancer and anti-inflammatory activity compared to curcumin [23, 24]. The curcuminoids, curcumin, DMC and BMC strongly inhibited βA-fibril formation in AD [25]. So in the present study, the neuroprotective effect of DMC against rotenone induced in vitro model of PD is analyzed by measuring the levels of reactive oxygen species (ROS), mitochondrial membrane potential (MMP) and the expressions of pro-apoptotic and anti-apoptotic indices in SH-SY5Y neuroblastoma cells.

## Methods

### Chemicals

Rotenone, DMC, thiobarbituric acid (TBA), MTT, 2–7-diacetyldichlorofluorescein (DCFH-DA), rhodamine 123 (Rh-123), heat-inactivated fetal calf serum (FCS), Dulbecco’s modified Eagle’s medium (DMEM), glutamine, penicillin–streptomycin, EDTA, and trypsin were acquired from Sigma-Aldrich (Bangalore, Karnataka, India. Anti- Bax, Bad, Bcl-xL, Bcl-2, caspase-3, caspase-6, and caspase-8, caspase-9, cyt-c (cytosol and mitochondria) anti-bodies were obtained from Cell Signaling Technology, Inc. (Beverly, MA, USA) and β-actin antibody were purchased from Santa Cruz Biotechnology, Inc., (Dallas, TX, USA). Anti-mouse and anti-rabbit secondary anti-bodies were purchased from Bangalore Genei Pvt. Ltd., (Bangalore, Karnataka, India). All other chemicals and biochemical used were of analytical grade.

### Neuronal cell culture

Human SH-SY5Y neuroblastoma cell lines were procured from National Center for Cell Science, Pune, India. The cells were grown in DMEM (Dulbecco’s modified Eagle’s medium) supplemented with 1% antibiotic/antimycotic and 10% FBS solution. Cultures were maintained in a humidified incubator at 37 °C in an atmosphere of 5% CO_2_ and 95% air. Cell culture medium was changed for every 2 days. DMC and rotenone were freshly made in dimethylsulfoxide (0.05%) for each experiment. DMC was added 2 h prior to rotenone treatment.

### Assessment of neuronal viability

About 3 × 10^3^ SH-SY5Y cells were seeded per well in a 96-well culture plate. To determine the toxicity of rotenone and DMC, cells in the medium were incubated with different concentrations of rotenone (5, 10, 50, 100, and 200 nM) and DMC (5 nM, 10 nM, 20 nM, 50 nM, 100 nM, 200 nM, 500 nM and 1 μM) for 24 h. To determine the neuroprotective effect of DMC, SH-SY5Y cells were pretreated with various concentrations of DMC (5 nM, 10 nM, 20 nM and 50 nM) for 2 h and then incubated with rotenone (effective dose) for 24 h. The cells were incubated with 5 mg/mL MTT for 4 h at 37 °C, after treatment with different testing agents. The medium was removed carefully after the incubation and the formazan crystals were dissolved in 150 μL of DMSO and absorbance of formazan reduction product was measured by spectrophotometer at 570 nm using a microplate reader. Four independent experiments were performed from each group [26]. Based on the results obtained from cell viability assay, the effective dose of DMC against rotenone toxicity was utilized to study the effect of DMC by assessing ROS, MMP, apoptosis, and apoptotic protein markers expression.

### Measurement of intracellular reactive oxygen species

A non-fluorescent probe, 2,7-diacetyl dichlorofluorescein (DCFH-DA), can penetrate into the intracellular matrix of cells, is oxidized by ROS to form fluorecent dichlorofluorescein (DCF). This method is used to estimate the levels of endogeneous ROS formation in control and experimental cells. After the Pre-treatment with DMC (50 nM) for 2 h, the cells (1 × 10^5^ cells/well in 6-well plates) were incubated with rotenone (100 nM) for 24 h and then incubated with 100 μL DCFHDA for 30 min at 37 °C and washed twice with PBS to remove the excess probe; Glucose enriched PBS is used for the suspension of cells and then transferred to a fluoroslide and visualized using a fluorescent microscope. Fluorescent measurements were done with excitation and emission filters set at 485 ± 10 nm and 530 ± 12.5 nm, respectively (Shimadzu RF-5301PC spectrofluorimeter) and the images were captured using fluorescence microscope [6].

### Measurement of mitochondrial membrane potential (MMP)

MMP changes were determined by the mitochondrial-specific, incorporation of a cationic fluorescent dye Rhodamine-123 (Rh-123). After treatment with DMC for 2 h and rotenone for 24 h as previously described, the cells (1 × 10^5^ cells/well in 6-well plates) were changed to fresh medium (100 μL) containing 1 μL of fluorescent dye Rh-123 (5 mmol/L) and kept for 30 min at room temperature (37 °C). The test cells were then collected, washed twice with PBS, and estimated by using blue filter (450–490 nm). Orange–red fluorescence is emited by polarized mitochondrion, and depolarized mitochondrion emits green fluorescence. Spectrofluorometer is used to measure the fluorescent intensity at 535 nm [27].

### Dual staining

Acridine orange (AO) and ethidium bromide (EB) fluorescent probes were used to analyze apoptosis by fluorescence microscopy. After treatment schedule as described in previous experiments, medium was removed from the plates; cells (1 × 10^5^) were washed with PBS twice and stained with 100 μg/mL of AO and EB stain. To remove excess dye, cells were incubated for about 20 min at room temperature and then washed with warm PBS. Fluorescence microscopy was used for the morphological studies and photographed. Further spectrofluorometer was used to measure fluorescence intensity at 535 nm [5].

### Immuno blotting

After the treatment schedule, SH-SY5Y cells (1 × 10^5^) in 6-well plates were harvested, washed with PBS, and lysed in 100 *μ*L lysis buffer (20 mM Tris-HCl, pH 7.4150 mM NaCl,1 mM EDTA, 30 *μ*g/mL apoprotein, and 1 mM phenyl methyl sulfonyl fluoride) followed by centrifugation (1000 x *g* for 5 min at 4 °C). The cytosolic fractions were saved and the pellets were solubilized in the mitochondrial lysis buffer (50 mM Tris pH 7.4, 150 mM NaCl, 2 mM EDTA, 2 mM EGTA, 0.2% Triton X-100, 0.3% NP-40, 100 *μ*M PMSF, 10 *μ*g/mL leupeptin, and 2 *μ*g/mL apoprotein) kept on ice and vertex for 20 min followed by pelleting at 1000 x *g* for 10 min at 4 °C in order to remove insoluble material. Protein concentration was quantified using Lowry et al. [28] and subjected to 10% polyacrylamide gel electrophoresis. The separated proteins were blotted onto a PVDF membrane using semidry transfer (BIORAD). 5% non-fat milk is used for blocking in TBS at 25 °C for 1 h, blots were probed with various antibodies: caspase-3, caspase-6, caspase-8, and caspase-9, cytochrome-c (Cyt-c) (cytosol and mitochondria), Bax, Bcl-2, BAD and Bcl-xL (1:1000) and *μ*-actin (1:2000). Horseradish peroxidase-conjugated anti-mouse or anti-rabbit IgG were employed as the secondary antibodies (1: 2000). Protein bands were visualized by enhanced chemiluminescence method with ECL-kit (GenScript ECL kit, USA) [29].

### Statistical analysis

Statistical analysis was performed by one-way analysis of variance followed by the Duncan’s multiple range test (DMRT) using Statistical Package for the Social Science software package version 12.0. Results were expressed as mean ± SEM for four experiments in each group. *P* < 0.05 was considered significant.

## Results

### Effect of DMC and rotenone on the survival of SH-SY5Y cells

**Fig. 2:**
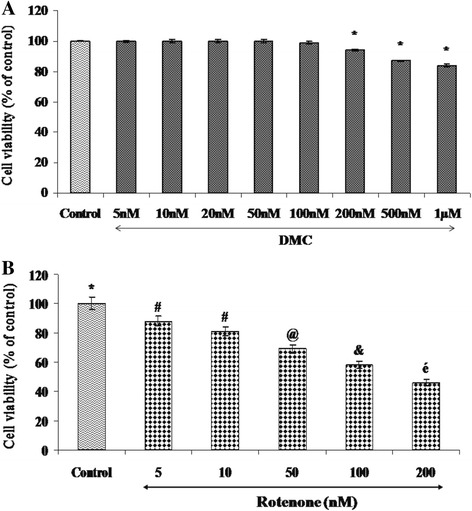
Effect of DMC on rotenone induced cytotoxicity in SH-SY5Y cells. **a** shows the dose dependent effect of DMC at various concentrations (5 nM, 10 nM, 20 nM, 50 nM, 100 nM, 200 nM, 500 nM and 1 μM). Up to 50 nM did not induce any toxicity after 24 h treatment, whereas slight toxicity was induced at 1 μM concentration. **b** shows the dose-dependent effect of rotenone (5, 10, 50, 100, and 200 nM) induced cell toxicity after 24 h. An approximately half-maximal inhibition of cell viability was obtained at 100 nM rotenone concentration. Values are expressed as the percentage of the untreated control and represented as mean ± SEM of four independent experiments in each group. Values not sharing a common superscript are significant with each other (*p*<0.05)

We first evaluated, whether DMC alone or rotenone alone treatment were toxic to SH-SY5Y dopaminergic cell line. Cells were treated with various concentrations of DMC (5 nM, 10 nM, 20 nM, 50 nM, 100 nM, 200 nM, 500 nM and 1 μM) and rotenone (5 nM, 10 nM, 50 nM, 100 nM and 200 nM) for 24 h and cell survival was determined by MTT assay (Fig. 2a and b). As shown in Fig. 2a, when exposed to DMC concentrations of 50 nM or lower, the viability of SH-SY5Y cells was the same as untreated control cells. However, a slight decrease of cell viability was observed with 100 nM and more significant toxicity was seen in 200 nM, 500 nM and 1 μM DMC treatment. Rotenone treatment (5 nM, 10 nM, 50 nM, 100 nM and 200 nM for 24 h) induced a dose-dependent reduction in cell viability with approximately LD50 observed at 100 nM (Fig. 2b). Consequently, cytotoxic induction with 100 nM rotenone for 24 h was used in the subsequent experiments.

### DMC protects against rotenone-induced cytotoxicity

**Fig. 3:**
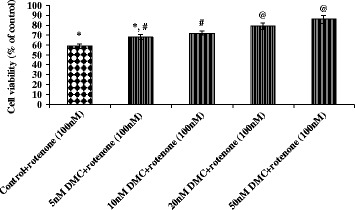
The protective effect of DMC (5, 10, 20 and 50 nM) against rotenone induced cell death was determined by MTT assay. Values are expressed as the percentage of the untreated control and represented as mean ± SEM of four independent experiments in each group. Values not sharing a common superscript are significant with each other (*p*<0.05)

The protective effect of DMC against rotenone induced toxicity with cell viability increasing to 86 ± 3.97% of control in the presence of 50 nM of DMC. So based on the dose-response data, the treatments of 50 nM DMC and 100 nM rotenone were chosen for further experiments (Fig. 3).

### DMC ameliorated rotenone-induced oxidative stress

**Fig. 4:**
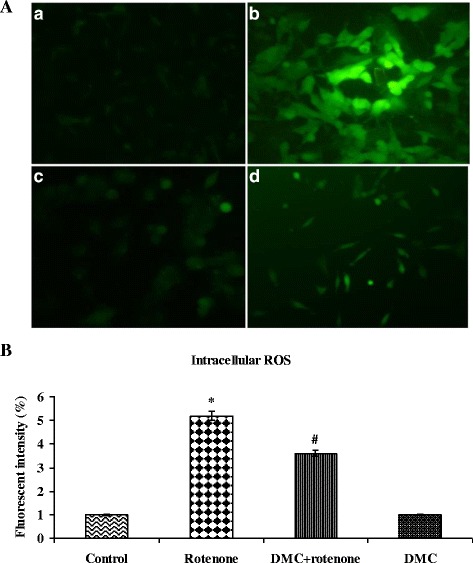
**a** DMC reduced ROS formation as stained by 1*μ*M CM-H2 DCFDA. **a** Photomicrograph showing the preventive effect of DMC (50 nM) against rotenone induced ROS generation. *a* Control, *b* rotenone, *c* DMC + rotenone and *d* DMC. **b** ROS levels were significantly increased in rotenone (100 nM) treated cells as compared to control cells, while DMC (50 nM) pretreatment significantly decreased the levels of ROS as compared to rotenone alone treated cells. Values are given as mean ± SEM of four independent experiments in each group. *p<0.05 compared to control and #*p*<0.05 compared to rotenone group (DMRT)

To determine the changes in intracellular ROS in human dopaminergic cells during rotenone-induced cell death and DMC mediated protection, ROS production in SH-SY5Y cells was measured using fluorescent dye DCF-DA. The levels of intracellular ROS markedly increased after treatment with rotenone. However, pretreatment with DMC (50 nM) significantly decreased rotenone induced ROS production (Fig. 4a and b).

### DMC prevents rotenone-induced reduction of the MMP

**Fig. 5:**
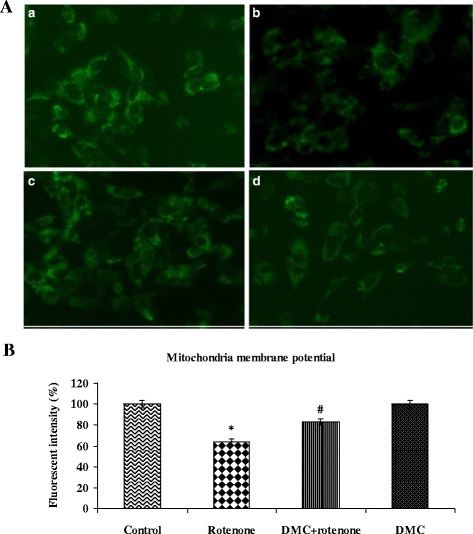
DMC stabilizes MMP as stained by Rh-123. **a** Photomicrograph showing the preventive effect of DMC (50 nM) against rotenone induced mitochondria membrane potential. *a* Control, *b* rotenone, *c* DMC + rotenone and *d* DMC. **b** Rotenone (100 nM) significantly decreased MMP, while cells that were pretreated with DMC (50 nM) significantly increased MMP. Values are given as mean ± SEM of four independent experiments in each group. *p<0.05 compared to control; #*p*<0.05 compared to rotenone groups (DMRT)

The MMP was rapidly reduced when SH-SY5Y cells were exposed to 100 nM of rotenone for 24 h, which was detected by the weakening of the fluorescence intensity of a mitochondrial specific probe, Rh-123. As compared to control cells, rotenone treatment increased the Rh-123 negative cells. Pretreatment with 50 nM of DMC protected cells against the rotenone induced lowering of MMP, decreasing Rh-123 negative cells (Fig. 5a and b).

### DMC ameliorated rotenone-induced apoptosis

**Fig. 6:**
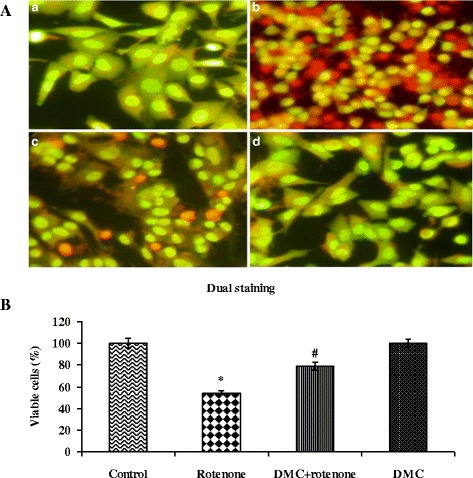
DMC protects SH-SY5Y cells against rotenone induced apoptosis. **a** Photomicrograph showing the antiapoptotic effect of DMC (50 nM) against rotenone at a concentration of 100 nM effective dose. *a* Control, *b* rotenone, *c* DMC + rotenone, and *d* DMC. **b** Rotenone (100 nM) treatment induced cell apoptosis compared to control cells; pretreatment with DMC (50 nM) suppresses these apoptotic features. Values are given as mean ± SEM of four independent experiments in each group. *p<0.05 compared to control and #*p*<0.05 compared to rotenone group (DMRT)

**Fig. 7:**
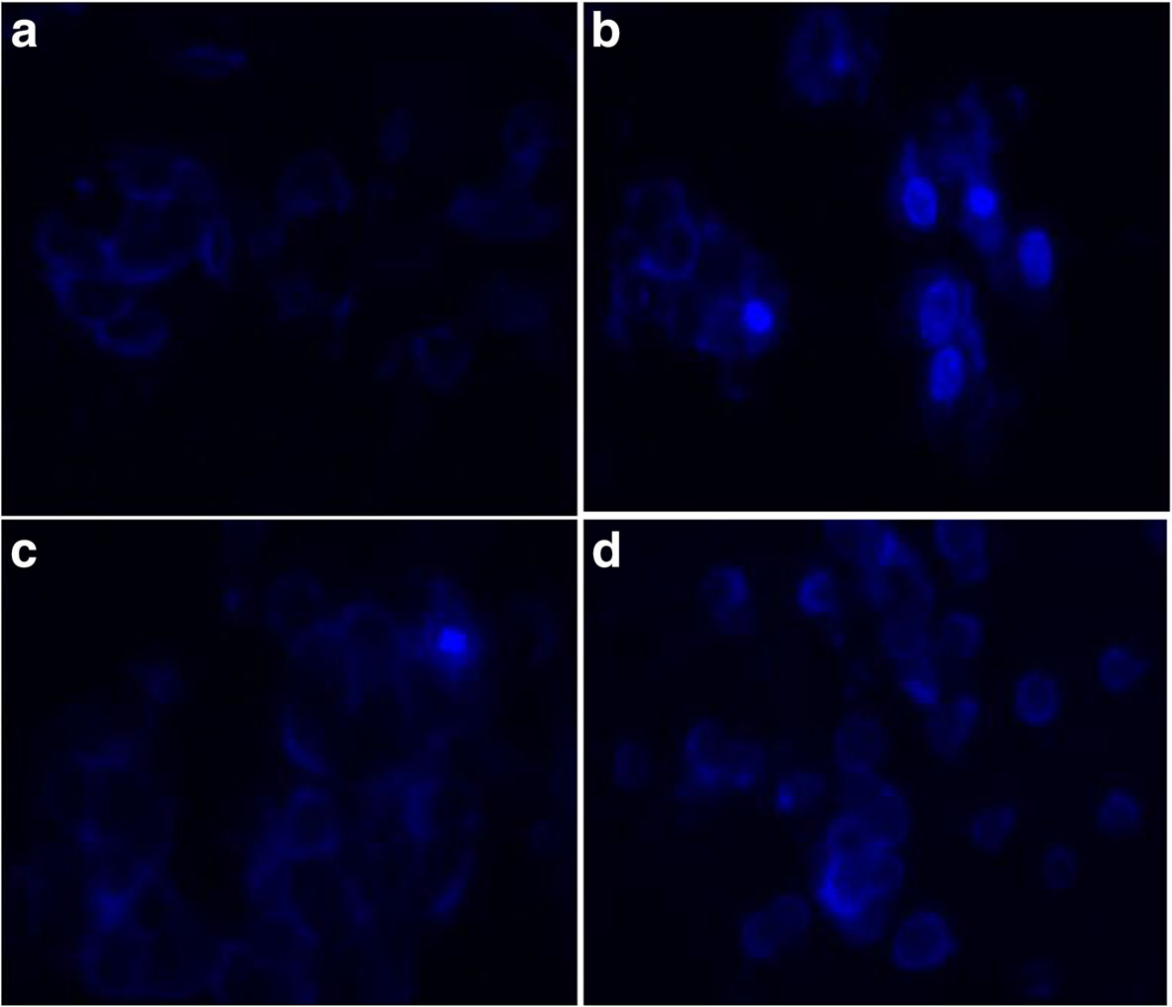
Nuclear morphology of SH-SY5Y cells stained with DAPI. Neuronal cells stained with DAPI showing the antiapoptotic effect of DMC (50 nM) against rotenone (100 nM). Nuclear condensation and/or fragmentation are indicator of apoptosis. **a** Control, **b** rotenone, **c** DMC + rotenone and **d** DMC. It is possible to observe some apoptotic cells in B, but not in the others groups

The rate of apoptosis was determined by double staining of rotenone and DMC treated SH-SY5Y cells, through AO and EB. Rotenone exposed cells revealed orange luminescent apoptotic body formation, when compared to control and treatment with DMC increased cell viability and decreased apoptotic cell death. Control cells which fluoresced brightly with green nuclei and normal morphology are showed in Fig. 6a and b. In Hoechst staining, treatment with 100 nM rotenone resulted in nuclear condensation and fragmentation. DMC pretreatment significantly protected the rotenone-induced nuclear damage (Fig. 7) due to its anti-apoptotic properties.

### DMC effect on rotenone induced proapoptotic and antiapoptotic gene expressions

**Fig. 8:**
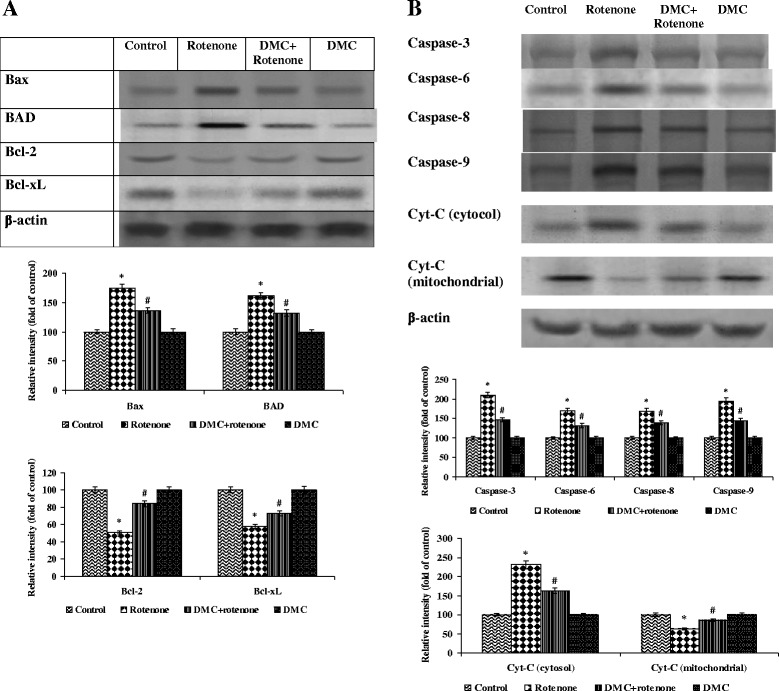
The effect of DMC on the expressions of apoptotic proteins. **a** and **b** show the expression of Bax, BAD, caspase-3, caspase-6, caspase-8, caspase-9 in mitochondria and Cyt-c in cytosol was increased while the expressions of Bcl-2, Bcl-xL and Cyt-c in mitochondria was significantly decreased by the rotenone treatment as compared with control. Pretreatment with DMC gradually restored the imbalanced expression profile of these proteins. Immunoblots are representative of at least four independent experiments. Values are given as mean±SEM in each group. *p<0.05 compared to control and #*p*<0.05 compared to rotenone group (DMRT)

To analyze the protective effect of DMC on rotenone-induced apoptosis, we assessed the expression of pro- and anti-apoptotic markers and Cyt-c release from the mitochondria in to the cytosol of cells. The expression of Bax, BAD, caspase-3, caspase-6, caspase-8, caspase-9 in mitochondria and Cyt-c in cytosol was increased whereas the distribution of Bcl-2, Bcl-xL and Cyt-c in mitochondria was significantly decreased by the rotenone treated group as compared with control. Pretreatment of cells with DMC gradually restored the excessive expression of these proteins (Fig. 8a and b).

## Discussion

At the time of initial diagnosis of PD, approximately 50% dopaminergic neurons in the nigro-striatal pathway have degenerated [3], and a large population of the remaining nigral neurons are affected by stress [30, 31]. So even in the in vitro model, it was important to employ concentrations of toxins that caused about 50% cell death, as the scenario resembles the situation at the time of initial diagnosis of PD. The data obtained from the MTT assay indicated that the rotenone (100 nM) treatment induced about 50% cell death, which is in consistent with previous studies demonstrating that exposure of cells to high concentrations of rotenone for a relatively short period of time (24 h) results in a mixed population of cells, in which some are healthy, some are no longer viable, and some are dead [5–7, 32]. Although DMC exerts its neuro-protective effect in a dose dependent manner, the maximum cell viability (86%) was obtained at (50 nM) concentration in MTT assay. This is chosen as an effective dose and used for further studies.

Normally the apoptotic features were measured in in vitro models using Dual, TUNEL, Hoechst 33,342 and DAPI staining. To confirm the anti-apoptotic effect of DMC against rotenone induced neurotoxicity on SH-SY5Y cells, we further performed the apoptosis assessment by dual staining. In dual staining, apoptotic cells uptake EB and emit red orange fluorescence, whereas AO is a DNA selective cationic dye that freely enters normal cell nuclei and emits green fluorescence. SH-SY5Y cells treated with rotenone emits more orange fluorescence than the control, while DMC pretreated cells emit more green fluorescence as compared to rotenone alone treated cells during dual staining. Results obtained from the MTT assay and dual staining technique, indicated that DMC inhibited the apoptosis induced by rotenone. Sirisidthi et al. [17] reported that the treatment of all the three curcuminoids (curcumin, DMC and BMC) increased the survival of rat PC12 and normal human umbilical vein endothelial cells (HUVEC) from amyloid β (1–42) insult, which corroborates our results.

Rotenone mimics the pathological features of PD in both in vitro and in vivo models by enhancing the overproduction of ROS [33], imbalance of cellular antioxidant systems [34], mitochondrial membrane depolarization [35], the formation and opening of the mitochondrial permeability transition pore [36], redistribution of Cyt-c [37], and eventually leading to cell death. The inhibition of complex I (also known as NADH:ubiquinone oxidoreductase; catalyzing the first step of electron transfer in the mitochondrial electron transport chain) by rotenone is accompanied with excess ROS formation and even a small level inhibition is sufficient to increase ROS production. Evidence from animal models suggests that rotenone induces oxidative effects that are responsible for some of the toxicity, and that these effects can be blocked by antioxidant therapy [38, 39]. Sirisidthi et al. [17] demonstrated that curcuminoids including DMC not only protects amyloid β (1–42) toxicity but also exhibit stronger antioxidative activity than vitamin-E. Dairam et al. [40] found that DMC is more potent in reducing lipid peroxidation than BMC. DMC has one methoxy group, while BMC has no methoxy groups. The presence of methoxy group on the phenyl ring might be responsible for this compound’s potent antioxidant property [41].

Oxidative stress is reported to be a primary mechanism of rotenone-induced degeneration of dopaminergic neurons [42] by inducing loss of MMP. It leads to the opening of permeability transition pores, or mega channels that has been identified as the first steps in the apoptotic process [43]. Apoptotic cell death, resulting from oxidative stress and diminished MPP, typically involves release of Cyt-c from the mitochondria to the cytoplasm. Rotenone treatment reduced membrane potential resulting in increased mitochondrial permeability and enhanced release of Cyt-c to cytosol, which leads to a decrease in its level in mitochondria fraction and a concomitant increase in cytosol [44]. Cyt-c is an important electron carrier in the mitochondrial respiratory chain and a death messenger in the cytosol to form apoptosome complexes with Apaf-1, dATP, and caspases 3 and 9 [45]. We found that DMC prevented rotenone induced MMP loss and cytosolic accumulation of Cyt-c, suggesting that neuro-protective effects of DMC are mediated in part by the preservation of mitochondrial function.

Anti-apoptotic (Bcl-2, Bcl-xL, Bcl-w, A1, and Mcl-1) and pro-apoptotic Bcl-2 family proteins (BAD, Bax, Bak, and Bok) are mainly responsible for the fine balance of apoptotic mitochondrial pathway regulation. The pro-apoptotic Bcl-2 family members Bax and Bak serve to permeabilize the mitochondrial outer membrane, allowing for release of Cyt-c, whereas the anti-apoptotic Bcl-2 family members including Bcl-2 and Bcl-xL function to inhibit these pro-apoptotic proteins [46]. Moreover BAD promotes apoptosis by forming heterodimers with Bcl-xL or Bcl-2 [47]. Akt phosphorylates BAD at serine 136 [48], whereas p9RSK, a downstream target of ERK1/2, phosphorylates BAD at serine 112 [49, 50]. Dephosphorylated BAD is localized to the mitochondria along with Bcl-2 and Bcl-xL, where it can induce apoptosis. When S112 and S136 of BAD are phosphorylated, BAD remain in the cytosol, as it is bound to 14–3-3 proteins rather than Bcl-2 or Bcl-xL [44]. This phosphorylated form of BAD does not promote apoptosis. We found that rotenone exposure increased the expressions of Bax and dephosphoryrated BAD and diminished the expressions of Bcl-2 and Bcl-xL, suggesting that neurotoxin would induce apoptosis. DMC showed its anti-apoptotic effect predominantly through the down regulation of Bax and BAD, and upregulation of Bcl-2 and Bcl-xL. Ahmed and Gilani, [51] indicated that DMC and curcumin protects neuronal cells from Aβ insult by enhancing the expressions of Bcl-xL and Bcl-2, where DMC was found to be more effective when compared with other constituents in the curcuminoid mixture. Moreover Liao et al. [52] demonstrated that all three curcuminoids including DMC activated extracellular signal-regulated protein kinase 1/2 (ERK1/2) in PC12 cells, thereby promoting the phosphorylation of BAD and inhibiting apoptosis.

Both the extrinsic (death receptor) and intrinsic (mitochondrial) apoptotic pathways are involved in the pathogenesis of PD. In intrinsic pathway, Cyt-c could form apoptosomes along with procaspase 9 and apoptosis-activating factor-1(Apaf-1), leading to the activation of caspase 9 and successive activation of caspase 3 [53]. Procaspase 9 binds to Cyt-c and Apaf to form an apoptosome by activating caspases 9, resulting in the subsequent proteolytic activation of the executioner caspases 3, 6 and 7, eventually resulting in apoptosis. In the extrinsic pathway, activation of caspases 8 results in proteolytic activation of the executioner caspases 3, 6, and caspases 7 resulting in apoptosis [54]. In the present study, expressions of caspases 3, 6, 8, and 9 significantly increased in rotenone exposed cells, which indicate that both the intrinsic and extrinsic pathways of apoptosis were activated. Animal studies led by Ahmed and Gilani, [51] showed that the oral administration of DMC decreased the levels of caspase-3 (the main executioner of both the pathways of apoptosis) in the hippocampus of Aβ (1–42) injected rats. Findings of our current study demonstrate that DMC pretreatment may suppress apoptosis, not only by regulating pro and anti-apoptotic indices but, by attenuating oxidative stress and mitochondrial dysfunction.

## Conclusions

This study demonstrates that curcumin is not the sole active pharmaceutical ingredient in turmeric, but that other constituents are also responsible for the neuroprotective effect of turmeric.

## Additional file

Additional file 1: The datasets supporting the conclusions of this article are included within the article. (DOCX 23 kb)

## Abbreviations

BAD: Bcl-2-associated death promoter
BCl-2: B-cell lymphoma 2
BCl-xL: B-cell lymphoma-extra large
Cyt c: Cytochrome-c
DA: Dopamine
DCFH-DA: 2,7-diacetyl dichlorofluorescein
DMC: Demethoxycurcumin
EDTA: Ethylenediaminetetraacetic acid
EGTA: (Ethylene glycol-bis(β-aminoethyl ether)-N,N,N′,N′-tetraacetic acid)
MMP: Mitochondrial membrane potential
MTT: Dimethylthiazol-2-yl)-2,5-diphenyltetrazolium bromide
PBS: Phosphate-buffered saline
PD: Parkinson’s disease
Rh-123: Rhodamine-123
ROS: Reactive oxygen species
