# Supplementary material for: Neuroprotective effect of Demethoxycurcumin, a natural derivative of Curcumin on rotenone induced neurotoxicity in SH-SY 5Y Neuroblastoma cells
Source: BMC Complement Altern Med. 2017 Apr 18;17:217. doi: 10.1186/s12906-017-1720-5 (PMC5395846; doi:10.1186/s12906-017-1720-5)
Supplement: Additional file 1: — The datasets supporting the conclusions of this article are included within the article. (DOCX 23 kb) [file 12906_2017_1720_MOESM1_ESM.docx]

Supplementary Materials

| MTT assay |  |  |  |  |  |  |
| --- | --- | --- | --- | --- | --- | --- |
|  | Control | Rot 5nM | Rot 10nM | Rot 50nM | Rot 100nM | Rot 200nM |
|  | 99.91 | 94.86 | 72.21 | 74.38 | 62.68 | 52.89 |
|  | 100 | 81.14 | 85.34 | 63.62 | 54.32 | 40.08 |
|  | 99.87 | 91.15 | 76.66 | 73.04 | 62.9 | 42.11 |
|  | 99.94 | 84.85 | 89.79 | 64.96 | 52.1 | 48.92 |
|  |  |  |  |  |  |  |
| Mean | 100 | 88 | 81 | 69 | 58 | 46 |
| SEM | 4.31 | 3.5 | 2.95 | 2.74 | 2.5 | 1.98 |
|  |  |  |  |  |  |  |

| DMC | Control | 5nM | 10nM | 20nM | 50nM | 100nM | 200nM | 500nM | 1µM |
| --- | --- | --- | --- | --- | --- | --- | --- | --- | --- |
|  | 99.43 | 100 | 99.15 | 100 | 100 | 100 | 91.12 | 93.87 | 90.47 |
|  | 99.84 | 98.85 | 99.2 | 99.55 | 99.2 | 99.67 | 97.67 | 93.79 | 78.54 |
|  | 100 | 100 | 100 | 99.34 | 100 | 98.99 | 99.9 | 81.67 | 86.13 |
|  | 99.73 | 99.64 | 100 | 99.43 | 100 | 98.89 | 87.13 | 83.89 | 81.17 |
|  |  |  |  |  |  |  |  |  |  |
| Mean | 100 | 100 | 100 | 100 | 100 | 99 | 94 | 87 | 84 |
| SEM | 3.31 | 3.64 | 3.98 | 4.31 | 3.98 | 3.8 | 3.49 | 3.46 | 2.78 |

| Rot + DMC | Rot (100nM) | 5nM DMC + Rot (100nM) | 10nM DMC + Rot(100nM) | 20nM DMC + Rot (100nM) | 50nM DMC + Rot(100nM) |
| --- | --- | --- | --- | --- | --- |
|  | 63.22 | 89.9 | 81.78 | 78.49 | 87.26 |
|  | 52.78 | 66.16 | 67.32 | 74.9 | 69.67 |
|  | 62.16 | 71.34 | 76.13 | 60.56 | 89.9 |
|  | 57.84 | 62.7 | 68.49 | 86.23 | 94.1 |
|  |  |  |  |  |  |
| Mean | 59 | 68 | 72 | 79 | 86 |
| SEM | 2.15 | 2.7 | 2.38 | 3.34 | 3.97 |

| ROS | Control | Rot | DMC+Rot | DMC |
| --- | --- | --- | --- | --- |
|  | 1.08 | 5.7 | 3.83 | 0.94 |
|  | 0.95 | 4.9 | 3.12 | 0.99 |
|  | 0.99 | 5 | 3.26 | 0.91 |
|  | 0.98 | 5.06 | 3.82 | 0.93 |
| Mean | 1 | 5.165 | 3.5 | 0.94 |
| SEM | 0.05 | 0.362 | 0.37 | 0.034 |

|  |
| --- |
|  |

| MMP | Control | Rot | DMC+Rot | DMC |
| --- | --- | --- | --- | --- |
|  | 100 | 75.5 | 89.5 | 100 |
|  | 99.99 | 63.78 | 68.2 | 99.99 |
|  | 100 | 68.1 | 81.9 | 100 |
|  | 99.95 | 49.3 | 82.4 | 98.97 |
| Mean | 99.98 | 64.17 | 83 | 99.84 |
| SEM | 0.023 | 11.03 | 10.81 | 0.513 |

|  |
| --- |
|  |

| Dual | Control | Rot | DMC+Rot | DMC |
| --- | --- | --- | --- | --- |
|  | 100 | 54.51 | 90.34 | 100 |
|  | 100 | 48.89 | 65.87 | 101.34 |
|  | 100 | 50.49 | 82.01 | 100 |
|  | 100 | 62.11 | 77.78 | 101.34 |
| Mean | 100 | 54 | 79 | 100.67 |
| SEM |  | 5.9 | 10.19 |  |

| **BLOTTING** |  |  |  |  |  |
| --- | --- | --- | --- | --- | --- |
| Bax | Control | Rot | DMC+Rot | DMC |  |
|  | 100 | 175.45 | 145.72 | 101.25 |  |
|  | 100 | 159.89 | 111.78 | 99.13 |  |
|  | 100 | 182.43 | 157.51 | 99.45 |  |
|  | 100 | 178.27 | 128.99 | 98.25 |  |
|  |  |  |  |  |  |
| Mean | 100 | 174.01 | 136 | 99.52 |  |
| SEM |  | 9.84 | 19.94 | 1.26 |  |
|  |  |  |  |  |  |
| Bad | Control | Rot | DMC+Rot | DMC |  |
|  | 100 | 177.45 | 147.89 | 101.25 |  |
|  | 100 | 146.98 | 123.63 | 102.19 |  |
|  | 100 | 157.68 | 118.11 | 99.30 |  |
|  | 100 | 161.89 | 138.37 | 98.42 |  |
| Mean | 100 | 161 | 132 | 100.29 |  |
| SEM |  | 12.63 | 13.61 | 1.732 |  |

| Bcl-2 | | Control | | Rot | | DMC+Rot | | DMC |  |
| --- | --- | --- | --- | --- | --- | --- | --- | --- | --- |
|  | | 100 | | 65.72 | | 96.98 | | 104.72 |  |
|  | | 100 | | 39.12 | | 79.64 | | 104.4 |  |
|  | | 100 | | 56.59 | | 70.71 | | 103.45 |  |
|  | | 100 | | 42.6 | | 88.67 | | 102.34 |  |
| Mean | | 100 | | 51 | | 84 | | 103.72 |  |
| SEM | |  | | 12.37 | | 11.34 | | 1.431 |  |
|  | |  | |  | |  | |  |  |
|  | |  | |  | |  | |  |  |
| Bcl-XL | | Control | | Rot | | DMC+Rot | | DMC |  |
|  | | 100 | | 78.38 | | 85.77 | | 102.34 |  |
|  | | 100 | | 51.78 | | 67.31 | | 101.23 |  |
|  | | 100 | | 61.17 | | 79.47 | | 102.34 |  |
|  | | 100 | | 40.67 | | 59.43 | | 105.34 |  |
| Mean | | 100 | | 58 | | 73 | | 102.81 |  |
| SEM | |  | | 15.96 | | 11.84 | | 1.76 |  |
| \|  \| \| --- \| | |  | | \|  \| \| --- \| \|  \| | |  | |  |  |
|  |  | |  | |  | |  |  |  |
|  | |  | |  | |  | |  |  |
| Caspase-3 | | Control | | Rot | | DMC+Rot | | DMC |  |
|  | | 100 | | 212.89 | | 155.4 | | 97.67 |  |
|  | | 100 | | 194.23 | | 126.52 | | 100.02 |  |
|  | | 100 | | 242.55 | | 161.7 | | 102.35 |  |
|  | | 100 | | 190.33 | | 140.38 | | 99.91 |  |
| Mean | | 100 | | 210 | | 146 | | 99.98 |  |
| SEM | |  | | 23.82 | | 15.76 | | 2.578 |  |
|  | |  | |  | |  | |  |  |
| Caspase-6 | | Control | | Rot | | DMC+Rot | | DMC |  |
|  | | 100 | | 157.62 | | 119.68 | | 101.10 |  |
|  | | 100 | | 179.28 | | 138.58 | | 103.56 |  |
|  | | 100 | | 182.38 | | 122.32 | | 104.45 |  |
|  | | 100 | | 160.72 | | 143.42 | | 102.00 |  |
| Mean | | 100 | | 170 | | 131 | | 102.77 |  |
| SEM | |  | | 12.63 | | 11.76 | | 1.33 |  |
|  | |  | |  | |  | |  |  |
|  | |  | |  | |  | |  |  |
|  | |  | |  | |  | |  |  |
| Caspase-8 | | Control | | Rot | | DMC+Rot | | DMC |  |
|  | | 100 | | 154.02 | | 116.97 | | 101.56 |  |
|  | | 100 | | 178.48 | | 146.45 | | 99.17 |  |
|  | | 100 | | 181.98 | | 137.55 | | 98.48 |  |
|  | | 100 | | 157.52 | | 155.03 | | 100.83 |  |
| Mean | | 100 | | 168 | | 139 | | 100.01 |  |
| SEM | |  | | 14.26 | | 16.32 | | 1.81 |  |
|  | |  | |  | |  | |  |  |
|  | |  | |  | |  | |  |  |
| Caspase-9 | | Control | | Rot | | DMC+Rot | | DMC |  |
|  | | 100 | | 209.13 | | 149.33 | | 101.87 |  |
|  | | 100 | | 212.16 | | 129.77 | | 99.66 |  |
|  | | 100 | | 184.78 | | 136.11 | | 100.11 |  |
|  | | 100 | | 170.13 | | 160.79 | | 98.21 |  |
| Mean | | 100 | | 194 | | 144 | | 99.96 |  |
| SEM | |  | | 20.11 | | 13.84 | | 2.01 |  |
|  | |  | |  | |  | |  |  |
| Cyt-C (cytosol) | | Control | | Rot | | DMC+Rot | | DMC |  |
|  | | 100 | | 242.18 | | 175.55 | | 105.23 |  |
|  | | 100 | | 198.11 | | 145.49 | | 98.79 |  |
|  | | 100 | | 226.38 | | 166.98 | | 99.51 |  |
|  | | 100 | | 217.58 | | 159.98 | | 101.41 |  |
| Mean | | 100 | | 221 | | 162 | | 101.23 |  |
| SEM | |  | | 18.37 | | 12.71 | | 1.18 |  |
|  | |  | |  | |  | |  |  |
|  | |  | |  | |  | |  |  |
| Cyt-C (mitochondrial) | | Control | | Rot | | DMC+Rot | | DMC |  |
|  | | 100 | | 50.76 | | 97.1 | | 101.45 |  |
|  | | 100 | | 66.93 | | 75.6 | | 99.13 |  |
|  | | 100 | | 59.07 | | 80.9 | | 97.98 |  |
|  | | 100 | | 75.24 | | 90.4 | | 102.87 |  |
| Mean | | 100 | | 63 | | 86 | | 100.35 |  |
| SEM | |  | | 10.49 | | 9.6 | | 2.21 |  |
